# An Overview of the Possible Exposure of Infants to Microplastics

**DOI:** 10.3390/life14030371

**Published:** 2024-03-12

**Authors:** Csilla Mišľanová, Martina Valachovičová, Zuzana Slezáková

**Affiliations:** 1 Institute of Nutrition, Faculty of Nursing and Professional Health Studies, Slovak Medical University, 833 03 Bratislava, Slovakia; martina.valachovicova@szu.sk; 2 Department of Nursing, Faculty of Nursing and Professional Health Studies, Slovak Medical University, 833 03 Bratislava, Slovakia; zuzana.slezakova@szu.sk

**Keywords:** microplastics, infants, breast milk, baby bottle, infant formula, feces

## Abstract

Microplastics are small plastic pieces with sizes less than 5 mm. They are becoming a global concern due to the potential risk to human health. The potential risks of microplastics may be greater for infants because they do not have sufficiently developed metabolizing enzymes, have less ability to remove microplastics, and have highly sensitive target organs. Infants should be breastfed for the first six months of life. Breast milk is considered to be the most complete and suitable source of nutrition. However, if breastfeeding during this period is not possible, it is necessary to use formulas designed for infant initial feeding. Infants may be exposed to higher levels of MPs through infant foods or plastic products. The aim of this study is to describe the possible sources of exposure to microplastics such as the human placenta, plastic feeding bottles, and toys as well as the presence of released microplastics in infant feces, breast milk, and infant formulas. There is still not enough data available for this study area. Therefore, it is necessary to pay increased attention to minimizing the negative effects of microplastics on human health.

## 1. Introduction

Plastic products entered the market in the 1950s. The production of plastics in Europe reached 58 million tons in 2014. More than 40% of plastic is single-use packaging. It is predicted that the amount of plastic waste will reach 13% by 2060 and will continue to increase. It is estimated that by 2040, about 250 million metric tons of plastic waste will enter the water system and about 460 million metric tons will enter the soil system. It is very difficult to imagine life without plastics, which have become part of our everyday life [1]. They are used in a lot of applications related to packaging, the automotive industry, aquaculture, fisheries, agriculture, building, furniture, transportation, personal care products, textiles, clothing, etc. [2]. Plastics are often used as materials in various industries due to their low production costs, easy transportation, low weight, and high resistance. Important environmental challenges include the necessity to reduce the amount of contaminants in the environment and eliminate and suppress their effects. Plastic pollution in all components of the environment such as water, air, soil, and biota, and their negative impact on human health are still a misunderstood research area [3,4].

The huge consumption of plastics and the associated large amount of plastic waste cause their constant release into the environment, where they can degrade into smaller pieces, microplastics (MPs), which are considered contaminants [5,6]. MPs are a synthetic material that contains a high proportion of polymers. MPs come from multiple sources, interact with different components of the environment, and have different routes of transport and transformation. Currently, microplastic pollution is an urgent worldwide problem due to environmental pollution. Statistics show that about 3 million tons of plastic is produced annually, and most of it is waste. Due to the influence of various external factors (UV radiation, weather effects, etc.), plastics degrade into smaller particles, MPs. As they are highly persistent substances that are not biodegradable and that accumulate in various environments, they represent a potential risk to human health. However, it should be kept in mind that MPs also serve as carriers of heavy metals, persistent organic pollutants that can have toxic effects on the human body [7].

The constant increase in plastics and their degradation products leads to their presence in components of the environment, including the food chain [2,8]. Pollution by MPs is a great concern because they can be transported over long distances and spread into soil, air, and water, but they are also found in various foods such as tap water, bottled water, seafood, honey, and salt. Air, indoor dust, and contaminated food and water have been considered the main sources of MP exposure. Recently, information about other possible sources of exposure to MPs, such as baby bottles, has been increasingly emerging, while studies on human stool, placentas, and fetuses provide evidence of exposure to MPs in infants and children. However, research in this area is still insufficient [9,10].

Scientific studies deal with their presence, distribution, and potential effects on the environment and living organisms. Monitoring and understanding the sources and pathways of MPs are key to developing strategies for their negative impact on the life of organisms on the planet [4].

The main goal of this review is to summarize the possible exposure of infants to MPs as well as the detection of possible released MPs in infant feces, breast milk, and infant formulas.

## 2. Characterization of Microplastics

A large amount of plastics, such as cosmetic products, personal care products, clothes, plastic bags, and bottles, enters the environment through various routes, for example, households, hospitals, and human, industrial, and agricultural activities. These products gradually degrade in the environment and turn into MPs depending on their molecular weight, chemical structure, crystallinity, additives, and functional groups. Although plastic degradation is a difficult process due to its properties, it is effective in the fight against plastic pollution [11,12].

MPs are usually defined as plastic particles and fibers smaller than 5000 µm. They are small enough to be easily overlooked, but they can have significant environmental and potential health consequences. MPs are divided into primary and secondary groups [13].

Primary MPs in the environment arise from plastic pellets or balls. They are used for commercial purposes and created by industrial activity in the production of personal care products, for example, shampoos, soaps, toothpaste, hair gel, etc. They are secondary to the degradation of plastic products by physical, chemical, and biological processes [14,15,16].

Secondary MPs arise as a result of the degradation of plastics (packaging, paints from various types of plastic products, fibers from textiles, etc.) by various physical, chemical, and biological processes including erosion, corrosion, photooxidation, and biological transformation. The most common way of MP formation is photodegradation [17,18]. Due to their small size, MPs can be consumed by marine animals, terrestrial organisms, and humans, which leads to concerns about their impact on ecosystems and human health.

Concerns about human health are growing as people are constantly exposed to MPs, in particular through the animal or plant food chain, food additives, beverages, and plastic packaging for food [17,18,19].

The mechanism of the degradation of primary microplastics to secondary ones is still not well known, but both groups have harmful effects on the environment and human health [20]. About 80% of MPs in the environment correspond to fibers and fragments [3,11,21,22]. The chemical formulas and structures of the most common primary MPs are listed in Table 1.

Due to the fact that plastics and MPs are hydrophobic and contain covalent bonds and functional groups resistant to attack, these substances are difficult to degrade. The degradation of plastics can take place mechanically, chemically, and physically but also biologically [23]. The different ways of degradation are shown in Table 2.

The degradation process of (micro)plastics can be influenced by their internal properties (composition, material, structure, etc.) as well as external environmental conditions (pH, temperature, humidity, catalysts, enzymes, etc.) [24].

**Table 2:** Types of abiotic and biotic degradation of microplastics.

Degradation	Degradation Methods		Consequences of Degradation	Ref.
	Photodegradation	Visible, infrared, UV light Reaction through light and reactive species involvement with a catalyst	- Chain scission - Cross-linking - Flexibility loss, color change - Oxidative decomposition - Generation of reactive oxygen species - Complete mineralization to H_2_O, CO_2_	[25,26,27,28]
ABIOTIC	Thermal degradation	1. Identification of MPs by pyrolysis gas 2. High-temperature-production H, CO, CH_4_, or fuel oil 3. As a pretreatment technique for MP degradation at low temperatures	- Conformational changes - Depolymerization of fragments - Breakage of polymeric backbone, molecular deterioration, changes in tensile strength, alteration of crystallinity, reduction in durability, cracks and color changes	[29,30,31]
		Hydrolytic degradation (hydrogen ions in acidic or alkaline media)	- Chain breakage - Surface corrosion of polyesters - Cross-linking - Chain breakage - Chain breaking, formation of products - Mineralization of MPs	[31,32]
	Chemical degradation	Oxidative degradation (heat, light, atmospheric oxygen)		
		Advanced oxidation processes		
		Bacterial degradation	- Enzymatic oxidation - Hydrolysis - Chain scission	[33,34,35]
		Fungal degradation		
BIOTIC	Biodegradation	Enzymatic biodegradation		[36]
		Combined biodegradation (by multiple bacteria)		[36]
		Algae degradation		[37]

Each type of polymer has different properties and, thus, they can be applied in various applications [10]. MPs have a different polymer structure composed of different monomers, which are responsible for physical or chemical properties [38]. The most commonly used is PE, which has been commonly used in packaging films, garbage and food bags, and many household items for a long time. PET is used for the production of water bottles, and caps are usually made from PP. Beverage cartons are usually made from PE, while PS is suitable for disposable food packaging with insulating properties [19]. According to Directive 2019/904 of the European Parliament and the Council, food and beverage containers made from PS have been restricted since 2021 [39]. MPs are present not only in the environment but also in households. Various disposable or reusable food containers, bottles, jars, cups, caps, and other types of plastic packaging are often used, which are usually made of PET, PP, PVC, and PS. Chemically, MPs have a polymeric structure and contain two main elements, carbon and hydrogen. Some other substances may contain bromide, chlorine, and oxygen. Chemical additives are typical for MPs. Their toxicity is related to the size of the MPs. It is true that the smaller the MP, the more toxic it is [12].

It is necessary to pay more attention to the toxic effects of MPs on the environment. Most publications are focused on the presence of MPs in the environment, methods of exposure to MPs, and their toxic effects on aquatic organisms. In terms of the toxicity of plastics and MPs, we can distinguish the following: 1. Self-toxic effects—these manifest in animals by stopping their growth, changing the structure of their intestinal microflora, and damaging their intestinal tissues. In addition, additives that are added to plastics (plasticizers, UV stabilizers, heat stabilizers, hardeners, biocides, pigments, etc.) are easily released into the environment in unstable conditions (for example, strong UV radiation, weathering, etc.) and damage different organisms in the environment. 2. The effect of load toxicity—due to the large specific surface and small particle size of MPs, there is strong adsorption of substances from the environment, such as polychlorinated biphenyls (PCBs), polycyclic aromatic hydrocarbons (PAHs), persistent organic pollutants (POPs), heavy metals, antibiotics, etc. These substances can indirectly support the production of toxic effects [40]. MPs absorbed by organisms in the environment can enter the human body through the food chain (for example, through fish) but also through canned food and bottled water and frequently cause various inflammatory reactions and induce oxidative stress [41].

MP exposure to the human body can occur through various routes, such as inhalation, through the skin, or by the ingestion of contaminated food [6,16]. The ingestion of contaminated food, which may contain dangerous substances such as PAHs or PCBs, occurs most often through the gastrointestinal tract [42]. Some MPs are excreted from the body through stool, in which PET has been observed. Its concentration in infant feces was found to be 10 times higher than in adult samples [43].

The problem of MP food contamination cannot be solved without taking environmental pollution into account. The accumulation of MPs has been detected in seas, oceans, surface and groundwater, soils, arctic snow and antarctic ice, and beaches. They are commonly spread through wastewater treatment plants, from which they can enter natural waters, groundwater, or terrestrial ecosystems [44,45,46,47].

People are in close contact with plastics and their degradation products, especially MPs, which have a negative impact on human health due to their complex physical and chemical properties (e.g., polymer, size, shape, charge). The vulnerability of children and pregnant women to these exposures must be taken into account [48].

It is very important to monitor the group (fetuses, newborns, infants, children) that is very sensitive to toxic substances because they do not have sufficiently developed metabolizing enzymes, have a lower ability to remove toxins, and have highly sensitive target organs [1]. 

MPs most often enter children’s bodies through toys, pacifiers, and contaminated food, but also by crawling on carpets and floors made of plastic. Baby bottles are an important source of MPs [49]. This will be discussed more in Section 3.1.

## 3. Sources of Exposure to Microplastics

Research on plastics is mostly focused on the main sources of exposure, namely, water and food, and the transfer of MPs to humans through the food chain, but there is little information on direct MP exposure through plastic products. Monitoring and understanding the sources and pathways of MPs are key to developing strategies for their negative impact on the life of organisms on the planet. MPs can enter food and drink from a variety of sources, including the environment, food packaging, and the manufacturing process. Some studies have shown that MPs can be present in food that is either packed in plastic packaging or that comes into contact with plastic materials in the production process [50].

Organizations such as the World Health Organization (WHO) [51] and the European Food Safety Agency (EFSA) [52] monitor this issue and carry out scientific studies to assess the possible risks associated with the presence of MPs in food. Some of the following studies prove exposure to MPs in infants and pregnant women.

A review article by Kadac-Czapska et al. [13] dealt in detail with current knowledge in the field of microplastics and their impact on human health. It characterized the routes of exposure, defines the sources of pollution, points out the most frequently occurring microplastics in food, and identifies the types of food contaminated with MPs.

Ragusa et al. [53] were the first to address the issue of the presence of MPs in the human placenta. They examined six placentas, four of which contained 12 MPs of various shapes. Based on these results in their research, Liu et al. [54] tested not only placentas but also infant feces, meconium, breast milk, and baby formula.

The exposure of fetuses to MPs through pregnant mothers is a potential risk to newborn children. Aghaei et al. [55] investigated the effect of polystyrene microspheres on the fetus and placenta in laboratory mice, with microplastic particles 50 nm–5 μm in size causing growth problems.

Hu et al. [56] also investigated the effects of exposure to polystyrene MPs in mice and found that these particles can adversely affect pregnancy by disrupting the immune system. 

Studies of animal models have found that nanopolystyrene particles can translocate from the maternal lung across the placenta to a fetus and its kidneys, heart, lungs, liver, and brain in late pregnancy [57,58,59]. In addition, using an ex vivo placental perfusion system, nanopolystyrene particles were found to be able to pass from the maternal uterine circulation to the fetal circulation via the placenta [60]. Recently, several microplastic fragments (5 to 10 μm in size) were found for the first time in four human placentas, suggesting that these MPs can pass into the placental tissue [61]. Based on these findings, there is growing concern about the potential risks of MPs to the embryo during pregnancy. However, there is the potential for the translocation of MPs from mother to fetus and the storage or accumulation of MPs in the fetus/embryo [59,60].

**Figure 1:**
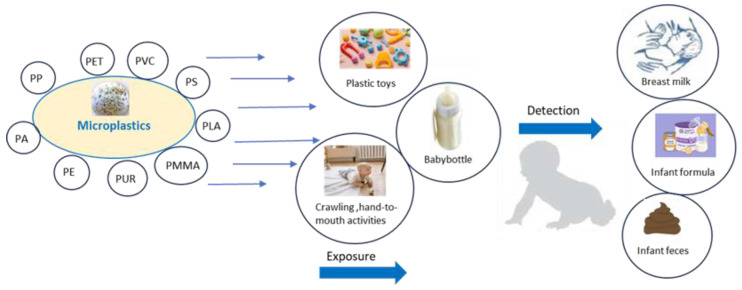
Sources of exposure to microplastics for infants.

Infants, children, and pregnant women constantly come into contact with plastics. The most studied areas of the occurrence of MPs are primarily air, food, and beverages, although exposure in infants can also occur through the placenta and breast milk and materials that come into contact with food [48,49]. These sources of exposure are still not well studied (Figure 1).

In addition to the human placenta [59], MPs have also been detected in children’s stool [62], but the sources of these plastics are relatively difficult to detect. Early exposures can occur through the placenta, during breastfeeding and the administration of infant formula, when a child breathes dust or licks and chews plastic toys and textiles, through baby bottles, etc. [49].

A review by Calatayud Arroyo et al. [1] described the interplay between the microbiota of the mother and the child—xenobiotics—as well as diet during pregnancy and in the perinatal period. Maternal exposure to metals, persistent organic substances, and food additives can cause changes in the microbiota of infants, and exposure can also result in the modulation of mother-to-child transmission of microorganisms during childbirth and breastfeeding [9]. A study focused on the initial evidence of MPs and additives in human amniotic fluid and placenta samples. The identified materials were chlorinated polyethylene and calcium zinc PVC stabilizers. The placenta acted as a partial barrier against the entry of MPs into the amniotic fluid and the fetus.

Similarly, tea bags are among the sources of exposure to MPs. During 5 min of exposing a tea bag to hot water (95 °C), 11.6 trillion MPs were released per cup of tea [61].

Given the high exposure and to avoid potential adverse effects, it is important to identify the main sources of microplastics to reduce exposure [63].

### 3.1. Microplastics in Baby Feeding Bottles

PP-based plastic products are often used for food preparation and storage. Such products also include lunch boxes and baby feeding bottles [64]. Baby feeding bottles and plastic packaging for baby food must be considered as potential sources of MPs. In [65], the possible exposure of infants to MPs through the contamination of infant formula in PP feeding bottles was investigated. A 21-day test was conducted on infants up to 12 months of age and in 48 regions in the central Amazon. The effects of water temperature, sterilization, and repeated use over a 21-day period on MP release levels were assessed. 

During the preparation of infant formula, feeding bottles are exposed to temperatures up to 100 °C in accordance with WHO guidelines. To assess the effect of temperature on MP release, they exposed bottles to deionized water at temperatures of 25, 40, 70, and 95 °C. The measured values of MP released were in the range of 14,600–4,550,000 particles per capita per day, depending on the region. The sterilization of bottles and exposure to high-temperature water during sterilization significantly increased the release of MPs. As a result of the prevalence of PP products used in the preparation of infant formula, the exposure of infants is higher than expected and, therefore, it is necessary to assess whether exposure to MPs at this level poses a risk to the health of infants [65].

MPs of irregular shapes released from baby bottles can cause increased cytotoxicity. A study by Xu et al. analyzed the thermal-oxidative processes of the aging of plastics and what kind of inflammatory response in human intestinal cells (Caco-2) can be induced by these irregular MPs. The exposure of intestinal cells to PP from a baby bottle triggered oxidative stress, which caused a decrease in glutathione levels, increased lipid peroxidation, and the release of reactive oxygen species. The levels of pro-inflammatory cytokines (IL-6 and TNFα), which are markers of the inflammatory process, increased and were even more intense during the disinfection of bottles with boiling water or when using microwave heating [66].

A Chinese study tested the release of MPs during the opening/closing process of a baby bottle, finding that 53–393 particles. mL^−1^ were released during 100 opening and closing cycles. In addition, they found that the type and brand of bottles, whether plastic or glass, affected the release of microparticles, suggesting that high-quality plastic and glass bottles release fewer microparticles and are more suitable for the health of infants and children [67].

Considering the results of this study, there is a growing need to focus more intensively on the potential of possible health risks arising from the use of baby bottles with PP contents.

Bisphenol A (BPA) is used in the production of PC plastics, which are used to make many products, including baby bottles. Despite the fact that most manufacturers of PC baby bottles declare that the products do not contain BPA, residual amounts of BPA have been detected in some products [68,69]. In a study, the authors tested 15 PC bottles with a clear label of BPA-free/safe/clear, which they subjected to a stress test (cleaning and rinsing). The results showed that some products contained a residual amount of BPA that did not exceed the tolerable daily intake (TDI) [68]. The EFSA established 50 g/kg/bw/day as the TDI of BPA based on non-observable adverse effect levels [70]. The amount of BPA increases with repeated use, cleaning, and sterilization. Therefore, it is recommended that parents change baby bottles more often and use a cleaning agent that reduces the risk of BPA leakage. The EU prohibited the use of PC plastics in infant feeding bottles. Since the safety of infants is of the utmost importance, parents should be aware of all the health risks associated with the use of plastic baby bottles [71].

### 3.2. Microplastics in Human Feces

Due to the properties of MPs, such as their persistence and slow degradation in the environment, their bioavailability for various organisms increases. The presence of MPs in an organism can cause the growth and reproduction of other organisms and achieve entry into the food chain. Most organisms use the process of excretion in feces to remove indigestible MPs. A study by Perez-Guevara et al. [62] from 2018 was the first study to identify microplastics in human feces. Studies that dealt with the extent of the contamination of feces with MPs began to appear more often. Schwabl et al. published a study where they assigned microplastics to nine different types of polymers in human feces. Currently, there is still little knowledge about the consequences of the behavior of MPs in feces that enter the environment. Research on MPs in feces continues to grow and it is important to understand the analytical methods available to identify and quantify MPs [72]. 

There are few studies that address the toxic effects of MP on humans, although some studies on laboratory animals have shown adverse health effects [73,74]. It is known that humans are exposed to MPs, but the degree of exposure is poorly understood. Some studies provide a dose range of exposure to MPs calculated using controversial empirical models; others report MP intake through various sources and pathways. But, research describing the burden of MPs on the human body is still lacking. One limitation is also the lack of reliable analytical methods for determining MPs in biological matrices. Since particles larger than 150 µm are reported to be excreted in feces, the determination of MPs in stool could indicate the degree of burden. The occurrence of MPs in the placenta has been proven, but there is no information about the presence of MPs in meconium or children’s stool. A USA study measured PET and PC, which are mainly used in the production of textile fibers, water bottles, and mobile phones, in samples of meconium and infant and adult feces. A depolymerization method followed by LC-MS/MS was used, with PET and PC doses assessed from fecal concentrations. A concentration of 36,000 ng.g^−1^ PET and 78 ng.g^−1^ PC was detected in the feces of six infants. In stool samples from eight adults, values of 2600 ng.g^−1^ for PET and 110 ng.g^−1^ for PC were detected in 10 stool samples. The concentration of PET in adults was an order of magnitude lower than in infants; the results for PC were approximately the same. High concentrations of MPs in infant feces can come from several sources of exposure, mainly the use of plastic products, such as baby feeding bottles, tableware, plastic teethers and toys, and plastic containers for baby food. A one-year-old child often puts plastic products in their mouth, sucks cloths, and crawls on carpeted surfaces. In the study, there was observed a higher content of MPs in infant feces than in adults. The first reason they cited was the possibility of the transmission of MPs from mother to child. Infants who consumed more than 600 mL of breast milk had a higher MP content than those who received a mixed diet. Infants who received a complementary diet (more than 50 g per day) had a lower MP content. From this, it follows that exposure to MPs probably occurs through breast milk. However, there may be more reasons. Some women use a breast pump and store their milk in breast milk storage bags, which can lead to milk contamination. Another source of exposure can be the use of baby bottles. MP contamination was detected in infants who consumed infant formula five times a day and infants who received breast milk from a bottle. Since MP content was detected in both milk and infant formula, the bottle was probably the potential source of exposure [54].

### 3.3. Microplastics in Breast Milk and Infant Formula

Nutrition plays an important role in the first few months of a child’s life for their physical and cognitive development. According to the WHO, a child should be exclusively breastfed for at least the first 6 months of life because breast milk is unique, complex, optimal, and irreplaceable in its composition. It represents the ideal form of nutrition for a newborn and is adapted to its needs. However, if the mother cannot breastfeed, it is necessary to replace breast milk with artificial milk intended for the initial feeding of infants. Although all breast milk substitutes differ from breast milk, it is necessary that these products provide a comparable rate of growth and metabolism observed in exclusively breastfed infants. For infants with specific health problems, formula must be adapted to eliminate or at least minimize the health problems [49].

Bisphenol analogs were detected in 62 breast milk samples and 54 infant formula samples. The median concentration (0.56 ng.g^−1^) of bisphenol F (BPF) was the highest in infant formula, while, in breast milk, the median concentration (0.01 ng.mL^−1^) of bisphenol S (BPS) was the highest [75].

A study by Liu et al. investigated the presence of MPs in 18 mother–infant pairs and assessed exposure in the placenta, meconium, infant feces, breast milk, and infant formula. Infant feces, breast milk, and infant formula samples were collected in the first 6 months of age. More than 74% of MPs had dimensions of 20–50 μm and were PA- and PU-dominated. For women, the sources of exposure could be cleaning products or toothpaste, while baby bottles and plastic toys could be the sources for infants. The total amount of released PA, PU, and PE in infants who also received complementary food > 50 g per day was significantly lower than in infants who did not. An interesting finding was that the way plastic toys were washed affected the amount of PET and PVC in the infants’ feces. In addition, higher consumption of breast milk in infants and a higher frequency of use of baby bottles caused an increase in the number of released MP particles. The number of MPs in feces was also higher in infants who sucked plastic toys [54].

Infant formula is packaged in plastic materials, which represent additional sources of MP exposure through dietary intake or oral activities in infants. Sealable disposable plastic bags that are sterile are often used to store freshly expressed breast milk. The US Centers for Disease Control and Prevention (CDC) recommend storing maternal medicine in these bags for 4 h at room temperature of 25 °C or 4 days in a refrigerator (4 °C). Given that these products are now often used and come into direct contact with breast milk, more detailed research on the risk of infant exposure is necessary [43,76]. For the study, the six most commonly used types of baby food packaging were selected, and the aim was to characterize the size, amount, and composition of microplastic particles released from these products. Large amounts of microparticles (<300 μm) and fragments (1–50 μm) were released, which were identified as PE, PET, and nylon-6 using Raman spectroscopy. The amount of released MPs was in the range of 0.61–0.89 mg.day^−1^ due to the average daily intake of breast milk for infants [43]. 

Baby food can be contaminated with various substances, such as inorganic and organic substances, drug residues, pesticides, and even MPs. Given that little is known about the extent of food contamination with MPs, there are knowledge gaps that prevent effective action on plastics. A Mexican study found an MP concentration in a milk sample of 6.5 ± 2.3 MPs.L^−1^. The study conducted in Mexico found the presence of MPs in 23 samples of branded milk with an average of 6.5 ± 2.3 particles.L^−1^. Evidence on children’s exposure to microplastics is quite limited. It is very important to know which products are contaminated and to what extent children are exposed to MPs. The most vulnerable period for MP exposure is the first few months of a child’s life because immunological, metabolic, cardiovascular, and neurobehavioral developmental processes are taking place. Children are more exposed to the environment than adults (crawling, hand-to-mouth activities) and eat and drink more per unit of body weight, which again increases exposure to environmental contaminants [77].

In a Polish study, 30 types of infant formula from pharmacies, drugstores, and supermarkets were tested. The products were intended for healthy babies as well as for infants with digestive problems. The infant formula samples came from six manufacturers. The contamination of infant formula is incompatible with food safety standards; the results of this study pointed to the presence of MPs in infant formula. Daily consumption in infants from birth to 6 months of age was 49 ± 32 MP particles.100 g^−1^. They assumed that one of the reasons for the high concentration of plastic particles in products is the type of packaging. The most common polymers identified were PE (63% of packaging) and PP (37% of packaging). Sources to which infants may be exposed are breast milk, plastic baby bottles, plastic toys, and disposable breast milk storage bags. In canned infant formula from the Chinese market, MPs were detected with fewer particles by one order of magnitude (4 ± 3 MPs.100 g^−1^) than products from the Polish market (51 ± 8 MP.100 g^−1^). Compared to the well-studied area of MP food contamination, the presence of MPs in baby food packaging is still not well studied [50]. A study by Liu et al. reported the release of large amounts of micro and submicron particles, flakes (<300 μm), and fragments (1–50 μm) when using commercially available disposable breast milk storage bags. Among the released particles, PE, PET, and nylon-6 predominated, which were identified by Raman spectroscopy. The weight of MPs released from the bags was 0.61–0.89 mg.day^−1^ with an average intake of breast milk. Infant formula is packaged in plastic material, which can be another source of MP exposure for infants through eating or food sources [76].

In a study by Li et al., it is stated that infant formula prepared in a PP bottle will release up to millions of MPs [54]. PET and PC were quantified in the feces of infants, the concentrations of which were significantly higher than in adults [44,54]. In infants who consumed more than 50 g of complementary food per day, the amount of total MPs, PA, and PU was significantly lower than in infants who did not receive complementary food. The method of washing plastic toys influenced the amount of released PET and PVC in infant feces. The more breast milk the infants drank, the greater the amount of MPs released into the feces. The amount of released MPs increased with the increasing frequency of drinking milk from feeding bottles. The amount of MPs in feces was higher in children who had a habit of sucking plastic toys. This study was the first study to comprehensively examine MP exposure in pregnant women, fetuses, and infants. The presence of MPs in breast milk and infant formula was detected. In children at an early age, exposure to MPs occurred through breastfeeding and the use of baby bottles and plastic toys. The content of PA was higher in meconium than in placentas [54]. Experimental studies investigating how MPs reach the embryo and fetus are still lacking. The increased content of MPs in meconium may be related to the long-term accumulation of MPs in meconium, which accumulates in the fetus from the 16th week of pregnancy and is not excreted until delivery. Apart from the mentioned study, only one other study found the MP contamination of breast milk. Infants’ exposure to MPs, especially PET and PC, in their daily diet is higher than that of adults. Children are more susceptible and come into contact with various plastic objects, such as plastic feeding bottles, toys, plastic dishes, etc. [78]. However, there is still a lack of information on neonatal exposure. Estimates so far are based on general food intake assumptions but do not take into account exposure in children with specific requirements. For infants who cannot be breastfed, the main component of the diet is powdered milk, which is packed in special packaging. However, there is no research to determine if powdered milk contains MPs. Contamination with MPs in milk powder does not only come from the milk powder but can also be released from the baby bottle and when the milk powder is brewing. In addition, MPs are commonly found in the air and in clothing. Few studies have correctly understood the need to consider all sources of exposure that may contaminate milk powder. The authors Zhang et al. conducted a comprehensive study of potential sources of contamination of powdered infant milk, namely, whether it comes from the packaging, powdered milk, baby bottles, or the preparation of the milk itself. They studied 13 different types of powdered milk with different types of packaging, milk preparation methods, and milk sources. There were more MPs in milk in boxes than that in cans. They assumed that the main source was the inner packaging of the boxes, which consisted of three layers: the outer special cardboard, the middle aluminum foil, and the inner PE layer. The exposure from powdered milk itself was low, exposure from baby bottles was 6.8 times higher, and exposure from milk preparation was 1.7 times higher [43].

## 4. Analytical Methods for the Identification and Quantification of Microplastics

Considering that MPs represent a great risk, it is necessary to put into practice developed general standard protocols (currently, they do not exist) that relate to the collection, characterization, and quantification of MPs. Analytical techniques relate mainly to sample collection and preparation and the identification and quantification of MPs. In the research field, there is still a deficit of standardized methods for the extraction of MPs from different samples, especially from sediment, air, and biological tissues [10,79].

Analytical methods for the identification and detection of MPs are described in various studies. An overview of some methods for the identification and detection of MPs is given in Table 3. 

Visual observation using a microscope is the most commonly used method because it does not require complex techniques and is based on the observation of particle size, shape, color, surface, and transparency. But, it appears to be an ineffective method of identification as the results obtained have often been overestimated or underestimated [10,79,90]. 

Infrared spectroscopy with Fourier transform (FTIR) is the most popular and widespread technique to identify the type of plastic of a MP found in the environment. It is a very precise, clean, and reliable method in which plastics can be easily distinguished from natural materials thanks to specificities that are shown in the spectra. Single-clean plastics have a specific structure and are therefore different from pure plastics. They do not have the same infrared spectra, which allows for unambiguous identification. FTIR is more sensitive for polar groups [79,90]. 

Raman spectroscopy is, together with infrared spectroscopy, the most widely used technique for the characterization of MPs. It can analyze Raman spectroscopy samples larger than 1 μm and allows for the tracking of very small differences in the molecular conformation of polymers, degree crystallinity with respect to amorphous regions, and stereoregularity of the polymer. Compared to FTIR spectroscopy, Raman spectroscopy achieves a better response of non-polar symmetric bonds [10,46,91]. 

Scanning electron microscopy (SEM) is suitable for MP identification and it is able to provide clear images of the vital physical properties of a particle. There is also the combination of SEM and X-ray dispersion spectroscopy (SEM-EDS), which is appropriate for determining the content of additives in plastics [92,93]. 

Pyrolysis-gas chromatography-mass spectrometry (Pyro-GC-MS) is a method for the characterization of MPs from the point of view of analysis of the degradation products of the given polymer. It consists of thermal degradation up to the pyrolysis of the polymer, which takes place in an inert atmosphere and breaks the chemical bonds of the polymer. Molecules with a lower molecular weight are formed, which are further separated by gas chromatography and detected by mass spectrometry. The sample is fragmented at a temperature between 500 and 1400 °C in the presence of helium and at low pressure, creating fragments that are introduced into the GC system. No sample pretreatment is required and the amount of sample can be within the range of 5–200 μg [91,94,95]. This methodo-logy makes it possible to simultaneously identify the type of polymer and organic filler and determine the chemical composition of MPs using spectral libraries and databases. It provides quantitative results with high accuracy, sensitivity, and selectivity. A small amount of sample is required and it allows for an analysis of the entire MP, not just its surface. The main disadvantage of this technique is that it is destructive and no information can be obtained about the color, number, size, or shape of the particle. It is not possible to analyze more MPs simultaneously as the analysis time is extended. Although this technique is highly effective in identifying different types of polymers, it is unable to distinguish between low- and high-density MPs [79]. 

Nuclear magnetic resonance (NMR) spectroscopy is used to identify MPs because it provides information about the molecular structure, crystallinity, and branching of monomers in copolymer compounds [10,91,95]. The principle of NMR is that the magnetic properties of atomic nuclei are used to obtain chemical information. NMR is used to calculate molar concentrations and monitor molecular changes in a sample, identify compounds present in samples, and study degradation [96]. 

Various new approaches are emerging in the field of detection and identification. For example, some authors describe fast identification using neural networks with one hidden layer, the so-called extreme learning machines (ELMs) [10,97]. A nonlinear vibration imaging technique—coherent anti-Stokes Raman scattering (CARS) microscopy—is used to identify ultrafine MPs [98]. A deep learning method has been developed, which uses a faster convolutional neural network model Faster R-CNN, where the residual network-50 (ResNet-50) and the pyramidal network module (FPN) are used [99].

## 5. Conclusions

The production of plastics was originally aimed at simplifying and improving human life, whether in industry, agriculture, or the home. However, this production has gotten out of control over the decades, and we have started to face unwanted consequences in the form of microplastics. These microparticles, which are created by the degradation of plastics, have spread to all areas of the environment, with a negative impact on human health and the overall ecosystem. Our review is based on scientific studies to clarify the extent of the presence of microplastics in various aspects of life, including breast milk, baby bottles, toys, and milk forms. On the basis of the finding that contact with microplastics occurs fromy young age through baby bottles and toys, it is found that there is a strong need for urgent measures. It is essential to examine their impact on children’s health and take measures to minimize exposure to these harmful particles. This significant problem requires immediate attention and action to ensure the healthy and safe development of children. Through further research and a systematic approach to tracking the sources of MPs, we will be able to create foundations for the development of effective strategies and minimize the negative impact of these microparticles on the environment and human health from an early age.

## Figures and Tables

**Table 1:** The most common primary microplastics.

Polymer	Chemical Formula	Chemical Structure	Polymer	Chemical Formula	Chemical Structure
PE	(C_2_H_4_)_n_	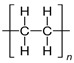	PET	(C_10_H_8_O_4_)_n_	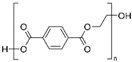
PP	(C_3_H_6_)_n_	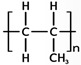	PPA	C_42_H_63_NO_7_	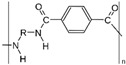
PVC	C_2_H_3_Cl	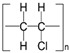	PC	(C_16_O_3_H_14_)_n_	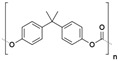
PS	(C_8_H_8_)_n_	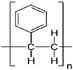	PU	(C_27_H_36_N_2_O_0_)	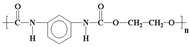
PES	(C_10_H_8_O_4_)_n_	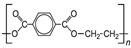	PLA	(C_3_H_6_O_3_)_n_	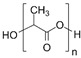
PA	(C_6_H_11_NO)_n_	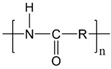	PMMA	(C_5_O_2_H_8_)_n_	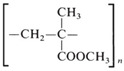
PAN	(C_3_H_3_N)_n_	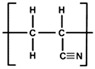			

PE—polyethylene; PP—polypropylene; PVC—polyvinyl chloride; PS—polystyrene; PES—polyester; PA—polyamide; PAN—polyacrylonitrile; PET—polyethylene terephthalate; PPA—polyphthalamide; PC—polycarbonate; PU—polyurethane; PLA—polylactic acid; PMMA—poly (methyl methacrylate).

**Table 3:** Examples of determination of polymers in various samples using different pretreatment techniques as well as methods for identification and detection.

Sample	Polymers	Sample Pretreatment	Method	Ref.
Water, sediment	PES, PA, PS	Catalytic wet peroxidation with H_2_O_2_, NaClO, and Fenton reagents,	SEM, FTIR	[80]
Sand	PE, PET, PS, PA, PVC	Wet peroxidation with H_2_O_2_ 5.3 M NaCl Filtration	μ-FTIR	[81]
Sand, sediment	PE, PS, PET, PP, PVC, Silicone, Nylon 6, Polyimide, Polysulfone	Drying, sieving Oxidation with H_2_O_2_ and Fenton reagents (H_2_O_2_ + Fe^2+)^	FTIR Raman spectroscopy	[82]
Indoor dust	PET, PA, PP, PE	Digestion with 30% H_2_O_2_ and ZnCl_2_ Filtration through metal filter, sonication with ethanol	LDIR LC-MS/MS	[83]
Surface water	PE, PP, PS, PVC, PET, PDMS, CPE	Oxidation with 30% H_2_O_2_ + 0.05 M FeSO_4_·7H_2_O 5 M NaCl Filtration	Pyr-GC/MS FTIR	[84]
Atmospheric aerosol Atmospheric deposition	PET, PC	Sieving, digestion, density separation, filtration, drying	Microscope, SEM, FTIR, Raman spectroscopy, LC-MS/MS	[85]
Surface water	PE, PP	Sieving, oxidation with 30% and 0.05 M Fe(II) 5 M NaCl Filtration, drying	SEM, FTIR	[86]
Marine fish (gastrointestinal tract)	PET, PP, PAN, PE, PVAc, PA, PS, PB, PC	Digestion with 10% KOH and 30% H_2_O_2_ Filtration, drying	SEM, FTIR	[40]
Water, sediment, tadpoles	PES, PP	Digestion with 30% H_2_O_2_ Filtration, drying	SEM, FTIR	[87]
Sea water	LDPE, PA, PET, PVC	Resuspension water SDS (150 g.L^−1^), ultrasonication, filtration	Raman spectroscopy	[88]
Indoor and outdoor air	PC, PE, PET, PS, PVC, PP		Raman spectroscopy	[89]

SDS—sodium dodecylsulfate; PDMS—polydmethylsiloxane; CPE—chlorinated polyethylene, PVAc—polyvinyl acetate; PB—polybutene; LDPE—low-density polyethylene.

## Data Availability

Not applicable.
